# Insulin-Related Lipohypertrophy: Lipogenic Action or Tissue Trauma?

**DOI:** 10.3389/fendo.2018.00638

**Published:** 2018-10-30

**Authors:** Anjana Barola, Pramil Tiwari, Anil Bhansali, Sandeep Grover, Devi Dayal

**Affiliations:** ^1^Department of Pharmacy Practice, National Institute of Pharmaceutical Education and Research, Mohali, India; ^2^Department of Endocrinology, Postgraduate Institute of Medical Education and Research, Chandigarh, India; ^3^Department of Psychiatry, Postgraduate Institute of Medical Education and Research, Chandigarh, India; ^4^Department of Paediatrics, Postgraduate Institute of Medical Education and Research, Chandigarh, India

**Keywords:** lipohypertrophy, type 1 diabetes, lipogenic action, tissue trauma, needle reuse, insulin analogs, insulin regimen, injection frequency

## Abstract

Lipohypertrophy has been suggested as an outcome of lipogenic action of insulin and/or injection-related tissue trauma. In a cross-sectional study, we evaluated the predictors of lipohypertrophy in 372 type 1 diabetes patients (mean age 17.1 years) receiving subcutaneous insulin with pen and/or syringes for ≥3 months. On examining injection sites with inspection and palpation technique, 62.1% patients demonstrated lipohypertrophy. Univariate analysis showed that gender, BMI, HbA1c, injection device, rotation, injection area, needle length, insulin regimen, and total daily dose of insulin were associated with lipohypertrophy (*p* < 0.05). Notably, the mean needle reuse was comparable in patients with or without lipohypertrophy (8.1 vs. 7.2, *p* = 0.534). In multivariate logistic regression, gender, HbA1c, TDD, injection devices, and needle length lost its significance. Further, injections over smaller area (≤8.5 × 5.5 cm) and non-rotation of sites were found to be strongest independent predictor of lipohypertrophy (*p* < 0.0005 for both) with increased odds of 23.2 (95% CI 9.1–59.2) and 6.3 (95% CI 3.4–11.9) times, respectively. Being underweight was also a significant independent predictor (odds ratio [OR] 13.0 [95% CI 2.2–75.2], *p* = 0.004). Compared to rapid plus long-acting analogs, regular insulin plus long-acting analogs and conventional premixed insulin users had 3.2 (95% CI 1.5–6.8, *p* = 0.003) and 4.6 (95% CI 1.4–15.7, *p* = 0.014) fold higher risk of lipohypertrophy (mean injection frequency 4.01 vs. 4.01 vs. 2.09, respectively). Sub-group analysis showed that lipohypertrophy was 79% less likely in patients with multiple daily injections (≥4) than twice-daily regimen (OR 0.21, *p* < 0.0005). Moreover, lipohypertrophy was reduced to half with bolus doses of rapid-acting insulin analogs than regular insulin (*p* = 0.003), even though mean injection frequency was comparable (4.01 vs. 3.93, *p* = 0.229). This difference was statistically insignificant for basal doses with NPH or long-acting analogs (*p* = 0.069). Therefore, injection area, rotation, BMI, and insulin regimen are the best predictors of lipohypertrophy and together could correctly identify lipohypertrophy status in 84.4% patients with excellent discrimination capability (AUC = 0.906, *p* < 0.0005). In conclusion, findings of our study suggest that delivering rapidly absorbed insulin analogs over large injection area along with greater split of total daily doses reduce insulin-induced lipogenesis and outplay tissue trauma added through frequent injections and needle reuse.

## Introduction

At present, the daily exogenous insulin replacement for the lifetime is the only available therapeutic option to manage majority of patients with type 1 diabetes mellitus (T1DM) (1). Repeated subcutaneous administration of insulin in the smaller area mount up the local lipogenic effect of insulin and injection-related tissue trauma, consequently, ensues lipohypertrophy in form of fatty lumps underneath the skin of that area (2). These lipohypertrophic swellings are not only unsightly but also may result into marked variability in insulin absorption, subsequently causing glycaemic excursions (3). A recent meta-analysis involving 26 studies has estimated 38% of overall pooled prevalence of lipohypertrophy in insulin-treated patients (4). Thus, mapping the predictors of lipohypertrophy is imperative to minimize this highly prevalent unwanted effect of insulin therapy.

Many researchers have examined the association of lipohypertrophy with age, gender, duration of therapy, body mass index, glycated hemoglobin, total daily dose of insulin (TDD), type of insulin, injection frequency, needle reuse, injection sites, and missing rotations (5–7). These observations have made important contributions to the current knowledge; however, there is a need of more studies because of numerous reasons. First, data regarding predictors of lipohypertrophy in T1DM were largely available from the limited studies (2, 8–12). Second, the relatively smaller sample sizes of these studies might have reduced their power to identify significant associations. Third, several researchers only employed univariate analysis, hence were unable to account the effect of other explanatory variables and confounders on the lipohypertrophy (8, 9). Fourth, some of these studies classified lipoatrophy as grade-3 lipohypertrophy (10), thereby invalidating the parameter estimates of lipohypertrophy. Lastly, after the introduction of newer insulin analogs, only one study involving T1DM patients examined the association of lipohypertrophy with type of insulin (12). Lipohypertrophy, being a primary outcome of lipogenic action of insulin, warrants systematic examination of its association with commonly used insulin classes and their administration frequencies. It is of particular interest, as multiple daily injections (basal-bolus regimens) have become the standard of care with introduction of newer insulin analogs.

Thus, present study aimed to examine the predictors of lipohypertrophy with particular emphasis on insulin regimens and types of insulin in a large sample of type 1 diabetes patients with appropriate statistical analysis.

## Materials and methods

### Participants

This cross-sectional questionnaire based study was conducted at Post Graduate Institute of Medical Education and Research (PGIMER) Chandigarh, India. A consecutive sample of 372 type 1 diabetes patients was recruited who agreed to participate in the study. The inclusion criteria were: (1) patients with history of diabetic ketoacidosis (DKA) and/or treated with insulin since diagnosis complemented with GAD-65 positivity and (2) at least 3 months of insulin administration with pen devices or syringes or combination of both. Exclusion criteria were (1) patients in partial remission (honeymoon phase) as identified by insulin dose-adjusted HbA1c (IDAA1c) < 9 (13); (2) patients prescribed with insulin regimens other than rapid-acting analogs plus long-acting analogs, regular insulin plus long-acting analogs, regular insulin plus Neutral Protamine Hagedorn (NPH) and conventional premixed insulins; and (3) patients with pregnancy.

Questionnaire was prepared by referring the relevant literature and consulting the experts. Patients who self-administered insulin were interviewed face to face; otherwise, responses were taken from the injection performing caregiver in the presence of the patient. All the participants were explained about the study protocol. In case of patients below the age of 18 years, we obtained written assent along with written parental or adult caregiver informed consent. For all the patients above the age of 18 years, a written informed consent was obtained. The study was conducted in accordance with the Declaration of Helsinki and approved by the PGIMER Institutional Ethics Committee.

### Parameters and procedures

Demographic information and current injection practice patterns were captured. The recorded parameters were age, gender, duration of diabetes, monthly family income, qualification and occupation of head of family, height and weight of the patient, injection administrator(s), glycated hemoglobin, type of injection device(s), needle length, extent of needle or syringe reuse, injections frequency and types of insulin (insulin regimen), total daily dose of insulin (TDD), injection site(s), injection area size measured within one side of site (for example, left or right side of the abdomen), rotation within injecting zone, and presence, type and location of lipodystrophy.

Real time update of Kuppuswamy socioeconomic status scale was used to measure the socioeconomic status of the patient. It is based on the composite score obtained from the occupation and qualification category of head of the family and monthly family income (14, 15). Principle earner of the family was considered as head of the family.

For adults, WHO recommended BMI cut-off points for Asians were used to categorize underweight (<18.5 kg/m^2^), normal (18.5–23 kg/m^2^), overweight (23.0–27.5 kg/m^2^) and obese >27.5 kg/m^2^) (16). For children between 5 and 18 years, revised Indian Academy of Pediatrics (IAP) growth charts were used to define overweight and obese category as adult equivalent of 23 and 27.5 cut-offs (17).

Glycated hemoglobin (HbA1c) values measured within past 2 weeks were documented. Needle use frequency was calculated by questioning “on an average how many insulin injections were administered with the single needle/syringe.” Size of needles and syringes was evaluated either based on self-report or looking into medical inventories. Insulin regimen, types of insulin, and total daily dose of insulin were noted from the medical records.

Size of the injecting zone was assessed using the standard size credit card (8.56 × 5.39 cm) (18), playing card (8.89 × 6.35 cm) and Indian postal card (14 × 9 cm) (19). Patients were shown these cards to recognize the size of their injecting area. Correct site rotation was defined as injections within any half or quadrant with spacing of at least 1 cm for the subsequent injection and then moving to next half or quadrant in the following week (20).

Presence of lipodystrophy at injection sites was ascertained with inspection and palpation techniques. Visible or palpable lump was indicative of lipohypertrophy, while depression at the injection site was suggestive of lipoatrophy (21). Lipohypertrophy was further confirmed with pinch maneuverer showing non-symmetric bilateral skin folds (22). Interviewer was not aware about the prior status of lipodystrophy because injection sites were checked only after the collection of all other data.

### Sample size calculation

We performed sample size calculations with Pocock's formula (n = Z^2^P(1 – P)/d^2^) using lipohypertrophy prevalence estimates of 41% from a previous study conducted in the same center (23). It predicted that 372 patients were needed to achieve 5% precision and 95% confidence interval.

### Statistical analysis

Predictors of lipohypertrophy were identified by examining data in sequential steps with SPSS version 20. The *P*-value <0.05 was considered as statistically significant.

In the first step, initial selection of variables explaining lipohypertrophy was done. Significant continuous and categorical variables were recognized with Mann-Whitney *U* test and Pearson chi-square test, respectively. Contingency tables showed that none of the categorical variables had a low expected count (<5 for 20% of cells) which ensured the suitability of Pearson chi-square test. In the second step, unadjusted odds ratio of each variable found to be significant in the first step were estimated with univariate binary logistic regression. In last step, we performed stepwise multivariate logistic regression analysis using backward selection approach and started with full model comprising all significant variables shortlisted in first step. Then we kept removing one insignificant variable at a time, until all the remaining variables stayed statistically significant that indicated the final model. Adjusted odds ratios estimated in the re-fitted final model were reported. Hosmer-Lemeshow goodness of fit test (HLGOF) and ROC analysis of the final model assessed its calibration and discrimination ability, respectively.

## Results

Of 372 patients enrolled in the study, 54.8% were males. The average age of patients was 17.1 years (range 5.0–50.6 years) with 5.6 years of mean duration of diabetes (range 0.3–36.7 years).

Insulin was administered with pen devices in 73.7% patients and by syringes in 20.2%. Remaining patients used combination of both (for example, bolus insulin with syringe and basal with pen or vice versa). The patients themselves administered insulin injections in 62.6% cases followed by patient-caregiver dyads (20.4%) and caregivers (16.9%). Basal-bolus therapy was prescribed to 87.4% (*n* = 325) patients. Of these, 62.2% (202/325) took rapid-acting insulin analogs and 37.8% (123/325) regular insulin for bolus doses. For the basal doses, long-acting insulin analogs were used in 91.4% (297/325) patients and NPH in 8.6% (28/325). Conventional premixed insulin regimen (2 to 3 injections) was recommended to 12.6% (*n* = 47) patients.

In the present study, lipodystrophy was observed in 62.6% of patients (*n* = 233). Of 372 patients, 229 exhibited lipohypertrophy, two lipoatrophy and two experienced both. Therefore, the total prevalence of lipohypertrophy was 62.1% (*n* = 231) and of lipoatrophy was 1.1% (*n* = 4). Total events were 379 as some patients developed lipohypertrophy at more than one site. With regard to the specific injection site, highest proportion of lipohypertrophic lumps were found at abdomen (200/316, 63.3%), followed by thigh (81/131, 61.8%) upper arm (85/138, 61.6%) and buttocks (5/10, 50.0%). Eight patients had lipohypertrophy at irrational sites (calf and forearm). When asked about injecting into lipodystrophic lesions, 60.8% patients indicated that they continued injecting into these areas.

Univariate analysis showed that age, duration of diabetes, socioeconomic status, injection administrator, needle reuse, TDD/kg body weight, and number of injection sites were not associated with lipohypertrophy (*p* > 0.05). Notably, the mean needle reuse was comparable in patients with or without lipohypertrophy (8.1 vs. 7.2, *p* = 0.534). Statistically significant association (*p* < 0.05) was attained between the lipohypertrophy and nine variables [gender, HbA1c, TDD, BMI, insulin delivery device, needle length, insulin regimen (injection frequency), injection area size, and injection rotation] (Tables 1, 2).

**Table 1:** Quantitative independent variables grouped based on lipohypertrophy status and the significance of observed differences.

**Variables**	**Total *n =* 372 Mean ±SD**	**Lipohypertrophy present (*n =* 231) Mean ±SD**	**Lipohypertrophy absent (*n =* 141) Mean ±SD**	**Significance (Mann-Whitney *U* test)**
Age (years)	17.1 ± 7.4	17.1 ± 7.8	17.2 ± 6.7	0.441
Duration of diabetes (years)	5.6 ± 5.3	5.7 ± 5.5	5.4 ± 5.1	0.381
HbA1c (%)	9.7 ± 2.6	10.0 ± 2.7	9.2 ± 2.4	0.007^a^
Frequency of needle reuse	7.8 ± 8.0	8.1 ± 8.9	7.2 ± 6.1	0.534
Total daily dose (TDD) of insulin (units)	40.7 ± 18.1	38.3 ± 16.9	44.8 ± 19.2	0.001^a^
TDD of insulin per kg bodyweight (units/kg)	0.97 ± 0.37	0.95 ± 0.37	0.99 ± 0.38	0.402
Insulin dose adjusted HbA1c	13.5 ± 3.0	13.7 ± 3.1	13.2 ± 2.8	0.097

a *Statistically significant*.

**Table 2:** Qualitative independent variables grouped based on lipohypertrophic status and the significance of observed differences.

**Variables**		**Total number (%)**	**Lipohypertrophy present number (%)**	**Lipohypertrophy absent number (%)**	**Significance (Pearson Chi square)**
Patients in study		372 (100)	231 (62.1)	141 (37.9)	0.008^a^
Gender	Male	204 (54.8)	139 (68.1)	65 (31.9)	
	Female	168 (45.2)	92 (54.8)	76 (45.2)	
Age	≤18 years	237 (63.7)	153 (64.6)	84 (35.4)	0.195
	More than 18 years	135 (36.3)	78 (57.8)	57 (42.2)	
Duration of diabetes	<1 year	49 (13.2)	28 (57.1)	21 (42.9)	0.898
	1–5 years	162 (43.5)	102 (63.0)	60 (37.0)	
	5–10 years	99 (26.6)	62 (62.6)	37 (37.4)	
	More than 10 years	62 (16.7)	39 (62.9)	23 (37.1)	
Socio economic status	Upper	24 (6.5)	13 (54.2)	11 (45.8)	0.135
	Upper middle	154 (41.4)	87 (56.5)	67 (43.5)	
	Lower middle	104 (28.0)	68 (65.4)	36 (34.6)	
	Upper lower	90 (24.2)	63 (70.0)	27 (30.0)	
BMI (kg/m^2^)	Underweight (<18.5)	57 (15.3)	42 (73.7)	15 (26.3)	0.001^a^
	Normal (18.5–23)	258 (69.4)	164 (63.6)	94 (36.4)	
	Overweight (23–27.5)	41 (11.0)	22 (53.7)	19 (46.3)	
	Obese (>27.5)	16 (4.3)	3 (18.8)	13 (81.2)	
Injection administrator	Patient	233 (62.6)	144 (61.8)	89 (38.2)	0.849
	Caregiver	63 (16.9)	41 (65.1)	22 (34.9)	
	Patient-Caregiver Dyad	76 (20.4)	46 (60.5)	30 (39.5)	
Injection device	Pen	274 (73.7)	155 (56.6)	119 (43.4)	0.001^a^
	Syringe	75 (20.2)	58 (77.3)	17 (22.7)	
	Pen and Syringe Combined	23 (6.2)	18 (78.3)	5 (21.7)	
Needle syringe length	4 mm	221 (59.4)	130 (58.8)	91 (41.2)	0.024^a^
	5 mm	18 (4.8)	7 (38.9)	11 (61.1)	
	6 mm	110 (29.6)	77 (70.0)	33 (30.0)	
	8 mm	23 (6.2)	17 (73.9)	6 (26.1)	
Needle reuse	1–3 times	106 (28.5)	62 (58.5)	44 (41.5)	0.840
	3–6 times	130 (34.9)	83 (63.8)	47 (36.2)	
	6–10 times	70 (18.8)	44 (62.9)	26 (37.1)	
	More than 10 times	66 (17.7)	42 (63.6)	24 (36.4)	
Insulin regimen (Mean injection frequency)	Rapid plus Long (4.01)	202 (54.3)	105 (52.0)	97 (48.0)	<0.0005^a^
	Regular plus Long (4.01)	95 (25.5)	64 (67.4)	31 (32.6)	
	Regular plus NPH (3.68)	28 (7.5)	21 (75.0)	7 (25.0)	
	Conventional Premixed (2.09)	47 (12.6)	41 (87.2)	6 (12.8)	
Number of injection sites	One Site	203 (54.6)	126 (62.1)	77 (37.9)	0.645
	Two Sites	106 (28.5)	63 (59.4)	43 (40.6)	
	Three Sites	63 (16.9)	42 (66.7)	21 (33.3)	
Injection area size (cm × cm)	Credit card size (8.56 × 5.39)	196 (52.7)	174 (88.8)	22 (11.2)	<0.0005^a^
	Playing card size (8.89 × 6.35)	113 (30.4)	46 (40.7)	67 (59.3)	
	Post card size (14 × 9)	63 (16.9)	11 (17.5)	52 (82.5)	
Systematic rotation	Non-followers	230 (61.8)	193 (83.9)	37 (16.1)	<0.0005^a^
	Followers	142 (38.2)	38 (26.8)	104 (73.2)	

a *Statistically significant*.

At first, we included all nine significant variables in the multivariate logistic regression. This model was statistically significant [$\chi_{\left(17\right)}^{2}$ = 234.730, *p* < 0.0005], explained 63.7% of the variance in the lipohypertrophy (Nagelkerke R^2^) and correctly classified 85.8% of cases. In this model, HbA1c, gender, TDD, injection device, and needle length lost their significance after adjusting for other variables. Next, we eliminated most insignificant variable one at a time starting from HbA1c (0.938), gender (*p* = 0.326), TDD (*p* = 0.224), injection device (*p* = 0.200), and needle length (*p* = 0.096) in a sequential manner. Even then, they remained insignificant in subsequent models and henceforth were dropped.

Lastly, multivariate logistic regression was performed to ascertain the effects of BMI, injection area size, injection rotation and insulin regimen (injection frequency) on the likelihood of lipohypertrophy. This re-fitted logistic model was statistically significant [$\chi_{\left(9\right)}^{2}$ = 222.171, *p* < 0.0005], well-calibrated (HLGOF *p* = 0.410) and had excellent discrimination quality (AUC = 0.906, *p* < 0.0005). All four variables remained statistically significant indicating the retention of this model as final. Correct case classification (84.4%) and explained variance were negligibly reduced (61.2%) when compared with starting model. Thus, elimination of the five variables viz. HbA1c, gender, TDD, injection device, and needle length from this final model did not affect its predictability.

Adjusted odds ratio estimated from the final model revealed that underweight patients had 13-folds increased odds for developing lipohypertrophy than the obese (*p* = 0.004). Further, patients injecting in area equivalent to credit card size were at 23.2 times higher risk for lipohypertrophy than post card size area users (*p* < 0.0005). Patients not performing injection rotation exhibited 6.3-folds more likelihood of lipohypertrophy than those adhering to rotation (*p* < 0.0005). Furthermore, regular insulin plus long-acting analogs users had 3.2 increased odds of developing lipohypertrophy than rapid plus long-acting analogs users (*p* = 0.003) with equivalent mean injection frequency (4.01 for both). Interestingly, patients treated with conventional premixed insulins had 4.6 times higher odds for lipohypertrophy than rapid plus long-acting analogs (*p* = 0.014). However, the mean injection frequency was lesser for conventional premixed insulins (2.09 vs. 4.01; Table 3). Sub-group analysis showed that lipohypertrophy was 79% less likely in patients with multiple daily injections (≥4) than the twice-daily regimen (unadjusted OR 0.21, *p* < 0.0005). Moreover, lipohypertrophy was reduced to half with bolus doses of rapid-acting insulin analogs than the regular insulin (*p* = 0.003) with comparable mean injection frequency (4.01 vs. 3.93, *p* = 0.229). This difference was statistically insignificant for NPH or long-acting analogs basal doses (*p* = 0.069).

**Table 3:** Unadjusted and adjusted odds ratio of significant variables explaining lipohypertrophy.

**Variables**		**Univariate binary logistic regression**		**Multivariate logistic regression**	
		**Unadjusted odds ratio (95% CI)**	**Significance**	**Adjusted odds ratio (95% CI)**	**Significance**
BMI (kg/m^2^) (Reference-obese >27.5)	Underweight	12.10 (3.03–48.56)	<0.0005	13.01 (2.25–75.24)	0.004
	Normal	7.56 (2.10–27.21)	0.002	11.76 (2.52–54.79)	0.002
	Overweight	5.02 (1.24–20.30)	0.024	7.25 (1.27–41.38)	0.026
Injection area size (Reference-post card)	Credit card	37.39 (17.02–82.16)	<0.0005	23.18 (9.07–59.24)	<0.0005
	Playing card	3.25 (1.53–6.88)	0.002	2.30 (0.97–5.47)	0.059
Rotation (Reference-followers)	Non-followers	14.28 (8.56–23.81)	<0.0005	6.31 (3.35–11.88)	<0.0005
Injection regimen (Reference-rapid and long acting analogs)	Regular plus Long	1.91 (1.14–3.18)	0.013	3.17 (1.49–6.78)	0.003
	Regular plus NPH	2.77 (1.13–6.81)	0.026	4.57 (1.35–15.54)	0.015
	Conventional premixed	6.31 (2.57–15.53)	<0.0005	4.62 (1.36–15.73)	0.014
Needle syringe length (Reference-4 mm)	5 mm	0.44 (0.17–1.19)	0.108	Dropped (0.096)^a^	
	6 mm	1.63 (1.00–2.66)	0.049		
	8 mm	1.98 (0.75–5.22)	0.166		
Injection device (Reference-pen)	Syringe	2.62 (1.45–4.73)	0.001	Dropped (0.200)^a^	
	Pen and syringe combined	2.76 (0.99–7.66)	0.051		
Total daily dose (units)		0.98 (0.97–0.99)	0.001	Dropped (0.224)^a^	
Gender (Reference-female)	Male	1.77 (1.16–2.70)	0.008	Dropped (0.326)^a^	
HbA1c (%)		1.13 (1.03–1.23)	0.007	Dropped (0.938)^a^	

a *These variables became insignificant after adjusting for other variables and was dropped in sequential order to obtain the final model*.

## Discussion

Our study demonstrates high prevalence of lipohypertrophy in patients with type 1 diabetes. It was predominantly observed in patients who did not use larger injection area, did not rotate insulin injection sites regularly, administered conventional premixed insulins, and were underweight. Further, needle reuse and frequent injections (basal-bolus regimen) did not increase the likelihood of lipohypertrophy.

In the present study, more than 60% of the patients developed lipohypertrophy, which is consistent with the recently reported estimate of 69.8% in Indian T1DM patients (12). Lipohypertrophy is relatively common in T1DM across the world with prevalence ranging from 28.7 to 76.3% (2, 9–12, 24). This wide range in prevalence may reflect differences in diagnostic accuracies. Prevalence recorded in the present study is higher than the previous estimates of 41% from our study center (23). It was not entirely unexpected, as with usual inspection and palpation techniques (21), we also used pinch maneuverer recommended by Gentile et al. (22) that could have improved diagnostic accuracy in the present study.

Correct injection site rotation is the most studied and emphasized approach for the prevention of this undesirable effect of subcutaneous insulin (2, 6, 24–26). Blanco et al. indicated that only 5% patients experienced lipohypertrophy who ensured proper rotation (24). Findings of Ji et al. survey shown that risk of exhibiting lipohypertrophy was increased by 8.4 times among rotation non-followers (6). Present study also reinforced the inevitability of correct site rotation in minimizing lipohypertrophy. In comparison to 83.9% of rotation-refraining patients, only 26.8% of rotation-complying patients developed lipohypertrophy.

Injection area size is an important determinant in the development of lipohypertrophy. It has been widely discussed that repeated injections in the smaller area could trigger lipohypertrophy (25, 27, 28). De Coninck et al. observed that 55.2% of the patients had lipohypertrophy who injected insulin in the area equivalent to credit card size, while it occurred in 42.9% of those utilizing the area of post card size (27). This association was more pronounced in our study revealing significant proportion of these patients having lipohypertrophy (credit card vs. post card, 88.8 vs. 17.5%).

Our and Hauner et al. (2) study underpin the association of low BMI with lipohypertrophy in patients with type 1 diabetes. In ultrasound examination, Perciun et al. demonstrated that remission of muscular and multilayer dystrophies in T1DM children did not happen even after sparing these areas from the insulin injections for 6 months. This finding is of particular interest because 35 of the 45 muscular and multilayer dystrophies were present in the underweight children (29). Conwell et al. also found the negative correlation between BMI Z-score and severity of dermatological changes in T1DM youth using continuous subcutaneous insulin infusion (30). Nevertheless, association of BMI and lipohypertrophy is discussed equivocally in literature. Ji et al. (6) and Saez-de Ibarra et al. (31) found high BMI to be associated with lipohypertrophy. Notably, their patients had BMI > 18.5 kg/m^2^ and were older (range 18–80 and 13–75 years, respectively) (6, 31) than the participants of the Hauner et al. (2) and our study (range 5–48 and 5.0–50.6 years, respectively). Consequently, both sets of studies were driven to measure only one end of the BMI and reached to opposite conclusions. Hence, BMI may have non-linear relationship with lipohypertrophy and both low and high BMI pose a risk. At least one study comprising wide aged sample (4–78 years) with all ranges of BMI supported this hypothesis (32).

Notably, patients receiving regular insulin plus long-acting analogs had 3.2-fold higher risk of lipohypertrophy than rapid plus long-acting analogs users with same mean injection frequency (4.01 for both). Relatively higher quantum of insulin monomers in rapid-acting analogs attributes to its faster absorption from subcutaneous tissue than human insulin. Thus, it can be hypothesized that rapid-acting insulin analogs with their improved pharmacokinetic actions might spare adipocytes from the lipogenic action as compared to conventional insulins. It is further supported by previous reports which suggest that faster absorption of insulin analogs reduces the stay of insulin in adipocytes and thereby provides a break from the local lipogenic action of insulin (33, 34). Findings of present study also showed that as compared to multiple daily injections of rapid plus long-acting analogs, risk of lipohypertrophy further augmented to 4.6 times with conventional premixed insulins (twice daily). It was contrary to the opinion that lipohypertrophy is the corollary of repeated tissue trauma caused by frequent injections (24, 25). Indeed, distributing total dose in small frequent bouts could minimize the extent of adipocytes' exposure to insulin. Similar trends were observed in study of Gupta et al. (12) for different insulin regimen classes, though inclusion of several insulin subgroups along with small sample size may have limited their ability to detect significant differences.

Our finding of higher HbA1c levels in patients with lipohypertrophy (mean difference = 0.8%) is in line with previous investigations (6, 25, 35), although the association was not statistically significant in present study. Besides, there are evidences suggestive of more episodes of unexplained hypoglycemia and glycaemic variability in presence of lipohypertrophy (12, 24, 25). Several pharmacokinetics and pharmacodynamics studies have confirmed that the blunted and varied absorption of insulin from the lipohypertrophic sites could cause glycaemic variability (3, 36, 37). Lipohypertrophy has also been reportedly associated with diabetic ketoacidosis in adolescents with type 1 diabetes (25, 38, 39). It has been shown that structured injection technique training could regress lipohypertrophy (7) and improve glycaemic control significantly (7, 40).

The clinical benefit received from the current recommendation of using needle just for once to prevent lipohypertrophy, is debatable. Several researches did not find significant difference in needle reuse between the patients with or without lipohypertrophy (5, 11, 41), consistent with our finding (mean frequency 8.1 vs. 7.2 times). In the beginning of twenty-first century, it was theorized that needle reuse causes deformity, dulling and bending of needle tips; and this added tissue trauma may instigate lipohypertrophy (42). Nevertheless, Kline and Kuhn observed no such damages to tips even after multiple insertions of needles through the rubber stoppers on insulin vials (43). Likewise, Puder et al. (44) and Berger et al. (45) also concluded that injecting into abdominal fat up to 5 and 10 times, respectively, did not lead to needle tip deformity. On the other hand, despite the statistically insignificant (*p* = 0.067) results, the pioneer European survey involving 1,002 patients accredited needle reuse as a contributor to lipohypertrophy (28). Furthermore, many subsequent studies unhesitatingly acknowledged the association of lipohypertrophy with needle reuse, only on the basis of univariate analysis (12, 27, 34, 46). Vardar et al. (26), Blanco et al. (24), Ji et al. (6), and Frid et al. (25) did employ the multivariate analysis in their investigations. While Vardar et al. (26) found that lipohypertrophy was associated with needle use more than once; the last three showed its association with lipohypertrophy at the needle reuse frequencies of 5, 7, and 10 times, respectively (6, 24, 25). Also, a meta-analysis performed by Zabaleta-Del-Olmo et al. demonstrated that in view of heterogeneity among existing scientific evidences, strong recommendation cannot be made regarding the needle reuse and presence of lipohypertrophy (47). Thus, the importance of needle reuse in development of lipohypertrophy has to be viewed with recurring extra expenses and time to comply with. To make a cost effective strategy, there is a high need to set the best cut-off value for the needle reuse frequency.

Therefore, in present study, injection rotation in the larger area had the greatest impact on lipohypertrophy reduction. In addition, patients receiving frequent injections (basal-bolus regimen) and rapid-acting insulin analogs experienced least events. Put together, injection over large area, insulin supply with rapidly absorbed insulins, and dosing in frequent split doses results into better distribution of total daily dose. It minimizes the accumulation of insulin in subcutaneous tissue and spares adipocytes from the lipogenic action of insulin. This notion has partly been discussed (33, 34); indeed, Bird et al. reported that one type 1 diabetes patient developed lipohypertrophy at insulin naïve site after 72 h of long-acting insulin administration but the same did not occur with short-acting insulin (48). Interestingly, needle reuse and frequent injections did not increase the likelihood of lipohypertrophy. In aggregate, lipohypertrophy seems to be a result of lipogenic action of insulin and not of tissue trauma caused by injections. Renold et al. also communicated the very same observation in their experiment (49).

The strengths of present study are large sample size, inclusion of only most commonly prescribed insulin regimens, and use of multivariate statistical analysis. It allowed for robust estimation of the important predictors of lipohypertrophy. To our knowledge, present study provided the first systematic examination of the association of lipohypertrophy with insulin regimens and types of insulin in T1DM patients using a large sample size.

As a limitation, we acknowledge that being single center study, generalization of findings may not be done. In addition, lipodystrophies were not examined through ultrasound imaging. Majority of patients did not perform self-monitoring of blood glucose, which has limited our ability to measure the glycaemic variability and hypoglycaemic episodes.

In conclusion, rotation of injections over the large area and use of rapid plus long-acting insulin analogs in patients with type 1 diabetes are the foremost and modifiable factors for minimizing insulin-related lipohypertrophy. Needle reuse and frequent injections did not contribute to lipohypertrophy.

## Ethics statement

The study was approved by the Institutional Ethics Committee (IEC) (Ref no: IEC-04/2016-377) and was performed in accordance with Helsinki Declaration. In case of patients below the age of 18 years, we obtained written assent along with written parental or adult caregiver informed consent. For all the patients above the age of 18 years, a written informed consent was obtained.

## Author contributions

AjB, PT, and AiB contributed to the conception and design of the study. AjB acquired and analyzed the data and drafted the manuscript. The work was supervised and monitored by AiB. PT, AiB, SG, and DD revised the manuscript and provided critically important intellectual inputs. Final version of manuscript was approved by all authors. All the authors agreed to be accountable for the content of the work.

### Conflict of interest statement

The authors declare that the research was conducted in the absence of any commercial or financial relationships that could be construed as a potential conflict of interest.
